# Supplementary material for: Time to breastfeeding cessation and its predictors among mothers who have children aged two to three years in Gozamin district, Northwest Ethiopia: A retrospective follow-up study
Source: PLoS One. 2022 Jan 21;17(1):e0262583. doi: 10.1371/journal.pone.0262583 (PMC8782324; doi:10.1371/journal.pone.0262583)
Supplement: S1 Questionnaire — (ZIP) [file pone.0262583.s002.zip › Questionnairs.docx]

## English version Questionnaires

Questionnaire code number: _____________

**Part-I: Socio-demographic predictors assessment questions (Baseline)**

| S.No | Questions | Responses | Skip |
| --- | --- | --- | --- |
| 101 | How old are you? | _____________years |  |
| 102 | What is your marital status at birth of the current child? | 1.Single 2.Married 3.Divorced 4.widowed 5. Others___________ |  |
| 103 | What is your educational status at birth of the current child? (the highest grade you completed?) | 1. cannot read and write 2. Able to read and write 3. Primary education 4. Secondary (9-12) 5. Certificate and diploma 6. Higher education and above |  |
| 104 | What is your husband’s educational status? | 1. cannot read and write 2. Able to read and write 3. Primary education 4. Secondary (9-12) 5. Certificate and diploma 6. Higher education and above |  |
| 105 | What is your occupation, that is, what kind of work do you mainly do during your current child breastfeeding process? | 1.Farmer 2.Housewife  3.Government employee  4.Private organizations employee  5.Private work(self-employee)  6. Student  7.Others________ |  |
| 106 | What is your family size? (Express number of children in bracket) | _________________ |  |
| 107 | How much is the wealth of the household? | ______________ | Fill wealth AQ |
| 108 | What is the sex of your child? | 1.Male 2.Female |  |
| 109 | Age of the child | __________________ |  |

**Part-II: Obstetric and Health service-related predictors assessment questions**

| 201 | Have you ever followed ANC (antenatal care) during your pregnancy of the current child? | 1. Yes 2. No | If no Skin to 203 |
| --- | --- | --- | --- |
| 202 | How many times did you receive antenatal care? | ___________ times |  |
| 203 | Total number of deliveries at the birth of the current child | ________________ |  |
| 204 | If >1 for Q203, How long was the birth Interval between the current child and his /her elder? | _______________months |  |
| 205 | How many babies did you deliver during the current child’s delivery? | _________________ |  |
| 206 | What is the birth order of the current child? | ____________________ |  |
| 207 | What was your mode of delivery for your current child? | 1. Normal 2. Cesarean section |  |
| 208 | Where did you give birth to your current child? | 1. your home 2. Health institution 3. Others _________ |  |
| 209 | Who assisted (attended) your delivery? | 1. Health professional 2. Traditional birth attendant 3. Relative/friend 4. Others_________ |  |
| 210 | Did you follow up with all immunization programs for the current child (Verify Certificate)? | 1. Yes 2. No |  |

**Part-III: Maternal breastfeeding-related predictors assessment questions**

| 301 | Have you ever breastfed before the current child? | 1. Yes 2. No |  |
| --- | --- | --- | --- |
| 302 | If yes to Q301, for how long did you breastfeed? | _____________ months |  |
| 303 | When did you start foods including liquids for your current child? | ___________months |  |
| 304 | Have ever used formula milk for the current child? | 1. Yes 2. No |  |
| 305 | Do you breastfeed the current child now? | 1. Yes 2. No |  |
| 306 | If No for Q306, how long after initiation should you had breastfed? | ________________months |  |
| 307 | Date of cessation of breastfeeding of the current child? | _________________dd/mm/yr. |  |

**Part-IV: Household Wealth Assessment Questions**

| S.no | Assessment Questions | Choices for response | | Response(circle) | | Skip |
| --- | --- | --- | --- | --- | --- | --- |
| 601 | What was the main source of drinking water for members of your household? |  | 1. Piped water to yard/plot 2. Common Piped water 3. Public dug well water 4. Plot/yard dug well with pump 5. Plot/yard dug well without pump 6. Water from protected spring 7. Water from unprotected spring 8. Surface water 9. Others | | |  |
| 602 | What did you usually do to make the water safer to drink? |  | 1. Add chlorine 2. Use water filter 3. Sprain through cloth 4. No safer agent | | |  |
| 603 | Did any member of this household have an account in a bank or other financial institution? |  | 1. Yes 2. No | | |  |
| 604 | Did you have your own household? |  | 1. Yes 2. No | | |  |
| 605 | Did this household have a separate room used as a kitchen? |  | 1. Yes 2. No | | |  |
| 606 | Did this household have a separate room for Animals? |  | 1. Yes 2. No | | |  |
| 607 | What are the main materials used for cooking in this household? |  | 1. Electricity 2. Charcoal/wood/grass 3. Kerosene 4. Animal dung | | |  |
| 608 | Main materials of the exterior walls of the dwelling? |  | 1. Stone with cement/blocket 2. Wood with Mud 3. Stone with mud 4. Others | | |  |
| 609 | Main materials of the floor of the dwelling? |  | 1. Earth/mud 2. Cement 3. Ceramic | | |  |
| 610 | Main materials of the roof of the dwelling? |  | 1. Corrugated iron 2. Thatch 3. Others | | |  |
| 611 | Did you have your own agricultural land? |  | 1. Yes 2. No | | |  |
| 612 | How many hectares of agricultural land did you have? |  | ---------------hcts (use local unit) | | |  |
| 613 | How much kuntal of the following cereals did you get annually? |  | 1. Teff------------ 2. Maize------------- 3. Barley/wheat-------------- 4. Bean/pea------------------ | | |  |
| 614 | Did this household have any of the following materials? | 1. Radio | | 1. Yes 2. No | |  |
|  |  | 1. Mobile | | 1. Yes 2. No | |  |
|  |  | 1. Water pump | | 1. 1. Yes 2. 2. No | |  |
|  |  | 1. Sewing Machine | | 1. Yes 2. No | |  |
|  |  | 1. Bed with mattress | | 1. Yes 2. No | |  |
|  |  | 1. Sewing Machine | | 1. Yes 2. No | |  |
|  |  | 1. Solar | | 1. yes 2. No | |  |
|  |  | 1. Cart | | 1. Yes 2. No | |  |
|  |  | 1. Bicycle | | 1. Yes 2. No | |  |
|  |  | 1. electricity | | 1. Yes 2. No | |  |
| 615 | Did any members of this household own any of the following livestock’?(If yes for the livestock’s please specify the number?) | 1. caw | | 1. yes_________________ 2. No | |  |
|  |  | 1. Horse | | 1. Yes_________________ 2. No | |  |
|  |  | 1. Donkey | | 1. Yes_________________ 2. No | |  |
|  |  | 1. Sheep | | 1. Yes________________ 2. No | |  |
|  |  | 1. Hen | | 1. Yes_______________ 2. No | |  |
|  |  | 1. Ox | | 1. Yes________________ 2. No | |  |
|  |  | 1. Mule | | 1. Yes______________ 2. No | |  |
|  |  | 1. Sheep | | 1. Yes____________ 2. No | |  |
|  |  | 1. Beehive | | 1. Y Yes ------------No | |  |
| 616 | How many rooms did have this household for sleeping? | | |  | ------------------ |  |
| 617 | Did this household have a domestic servant? | | |  | 1. Yes 2. No |  |
| 618 | How much was your household income per month? | | |  | ______ birr |  |
| 619 | Did you have cow milk in your household for the last two years? | | |  | 1. Yes 2. No |  |

**Thank you!!**

## **Amharic version Questionnaires**

**መጠይቆች**

የመጠየቅ ቁጥር(ኮድ)-------------------------

**ክፍል አንድ፡ የማህበራዊ አኗኗር ሁኔታ የዳሰሳ መጠይቆች (ህጻኑን በወለዱነት ግዜ)**

| ተ.ቁ | ጥያቄዎች | የመልስ አማራጮች | ይለፉት |
| --- | --- | --- | --- |
| 101 | እድሜዎ ስንት ነዉ? | ________________ዓመት |  |
| 102 | የጋብቻዎ ሁኔታ | 1. ያላገባች 2. ያገባች 3. የፈታች 4. ባል የሞተባት 5. ሌላ ከሆነ ይገለጽ---- |  |
| 103 | የትምህርት ደረጃዎ ( ክፍል የጨረሱት) | 1. ማንበብ እና መፃፍ የማትችል 2. ማንበብ እና መጸፍ የምትችል 3. የመጀመሪያ ደረጃ ትምህርት 4. ሁለተኛ ደረጃ (9-12) 5. ሰረተፈኬት እና ዲፕሎማ 6. ከፍተኛ ትምህርት እና ከዚያ በላይ |  |
| 104 | የባለቤትዎ የትምህርት ደረጃ( ክፍል የጨረሱት) | 1. ማንበብ እና መጻፍ የማይችል  2. ማንበብና መጻፍ የሚችል  3. የመጀመሪያ ደረጃ ትምህርት  4. ሁለተኛ ደረጃ ትምህርት  5. ሰርተፊኬትና ዲፕሎማ  6. ከፍተኛ ትምህርትና ከዚያ በላይ |  |
| 105 | የተሰማሩበት የስራ መስክ / ዋና ስራዎ) ምንድን ነው | 1. አረሶ አደር 2. የቤት እመቤት 3. የመንግስት ሰራተኛ 4. የግል ድርጅት ሰራተኛ 5. የራሴን ስራ እሰራለሁ 6. ተማሪ 7. ሌላ ካለ ይገለፅ------- |  |
| 106 | የቤተሰብ ብዛት ስንት ነዉ(የልጆችን ብዛት ይግለጹ) | -------------------------------- |  |
| 107 | የቤተሰቡን የሀብት መጠን ይግለፁ | -------------------- | ቤተሰብ ሀብት መለኪያ |
| 108 | የህጻኑ/ኗ ጾታ ምንድን ነዉ? | 1. ወንድ  2. ሴት |  |
| 109 | የህጻኑ እድሜ | ----------------------- |  |

**ክፍል ሁለት፡ የስነ ተዋልዶ ጤናና የጤና አገልግሎት ዳሰሳ መጠይቆች**

| 201 | የአሁኑን ህፃን ነፍሰጡር በነበሩበት ግዜ የቅድመ ወሊድ ክትትል አድርገዉ ያዉቃሉ | 1. አዎ 2. አላደረገሁም |  |
| --- | --- | --- | --- |
| 202 | መልሱ ለ201 አዎ ከሆነ ስንት ግዜ አደረጉ | ------------------------ |  |
| 203 | እስካሁን ስንት ልጆች ወልደዋል | ------------------------- |  |
| 204 | መልሱ ለ203 ከ1 በላይ ከሆነ በመካከላቸዉ ምን ያህል እድሜ ይበላለጣሉ | ----------------------- |  |
| 205 | ከአሁኑ ኀፃን ጋር ስንት ልጆችን ባንድ የምጥ ግዜ ወለዱ | ------------------------- |  |
| 206 | ይህ ኅጻን ስንተኛ ልጅዎ ነዉ | ------------------------ |  |
| 207 | የአሁኑን ኅጻን የወለዱት የት ነበር | 1. ቤት 2. የጤና ተቋም 3. ሌላ ካለ ይገለፅ-- |  |
| 208 | የአሁኑን ኅፃን የወለዱት በምን መንገድ ነበር | 1. አምጦ በመዉለድ 2. በማህጸን ቀዶ ጥገና |  |
| 209 | የአሁኑን ህፃን በወለዱበት ግዜ ያዋለደዎት ማን ነበር | 1. የጤና ባለሙያ 2. የልምድ አዋላጅ 3. ዘመድ/ጉድኛ 4. ሌላ ካለ ይገለፅ- |  |
| 210 | ለአሁኑ ህፃን ሁሉንም የክትባት ፕሮግራሞች ተከታትለዉለታል (ሰርተፍኬት ያረጋግጡ) | 1. አዎ 2. አልተከታተልኩም |  |

**ክፍል ሶስት፡ ከእናቶች ጡት ማጥባት ጋር የተያያዙ ዳሰሳ መጠይቆች**

| 301 | ከአሁኑ ህጻነን በፊት ጡት አጥብተዉ ያዉቃሉ | 1. አዎ 2. አላዉቅም |  |
| --- | --- | --- | --- |
| 302 | መልሱ አዎ ከሆነ ለ301 ለምን ያህል ግዜ አጠቡ | -------------ወራት |  |
| 303 | ያሁኑን ህጻን ከጡት በተጨማሪ ሌላ ምግብ/ፈሳሽ መስጠት የጀመሩት መቼ ነበር | --------------ወራት |  |
| 304 | ለአሁኑ ህጻን በፋብሪካ የተቀነባበረ የህጻናት ወተት ሰጥተዉት ያዉቁ ነበር | 1. አዎ 2. አላዉቅም |  |
| 305 | አሁን ህፃኑን ጡት እያጠቡት ነዉ | 1. አዎ 2. አይደለም |  |
| 307 | መልሱ አይደለም ከሆነ ለ306 ጡት ማጥባት ከጀመሩበት ግዜ አንስተዉ ለምን ያህል ግዜ አጠቡት | --------------ወራት |  |
| 308 | ጡት ማጥባት ያቁአረጡበት ቀን፣ወር እና ዓመት ይንገሩኝ | -ቀን--ወር--ዓመት |  |

**ክፍል አራት፡ የቤት ሃብት ዳሰሳ ጥናት መጠይቆች**

| ተ.ቁ | የደሰሳ ጥያቄዎች | የምላሽ ምርጫዎች | ምላሽ (ክበብ) | **ይለፉት** |
| --- | --- | --- | --- | --- |
| 601 | ለቤተሰብዎ አባላት የመጠጥ ውሃ ምንጭ ከየት ነበር? |  | 1. የግቢ ዉስጥ የቧንቧ ውሃ 2. የጋራ የቧንቧ ውሃ 3. የጋራ የጉድጓድዉሀ 4. የግቢ ዉስጥ የጉድጓድ ዉሃ ከነፓንፑ 5. የግቢ ዉስጥ የጉድጓድ ዉሃ ፓንፕ የሌለዉ 6. የተጠበቀ የምንጭ ዉሃ 7. ያልተጠበቀ የምንጭ ዉሃ 8. የወለል ዉሃ 9. ሌሎች |  |
| 602 | ብዙውን ጊዜ ውሃው ምቹ የመጠጥ ዉሃ እንዲሆን ለማድረግ ምን ያደርጉ ነበር? |  | 1. ማፍላት 2. ክሎሪን መጨመር 3. በጨርቅ ማጣራት 4. የዉሃ ማጣሪያ ማሽን መጠቀም 5. እንዲቆይና እንዲጠል ማድረግ 6. ምንም የለም |  |
| 603 | ከዚህ ቤተሰብ አባል ዉስጥ የባንክ ወይም በሌላ የገንዘብ ተቋም ውስጥ አካውንት ያለው ነበር? |  | 1. አዎ 2. የለም |  |
| 604 | የራስዎ ቤት ነበረወት? |  | 1. አዎ 2. የለም |  |
| 605 | ይህ ቤተሰብ እንደ መብሰያ ቤት የሚያገለግል የተለየ ክፍል ነበረዉ? |  | 1. አዎ 2. የለም |  |
| 606 | ለእንስሳት መኖሪያ የሚሆን የተለየ ቤት ነበረዉ |  | 1. አዎ 2. የለም |  |
| 607 | ለማብሰያ ምን ዓይነት ነዳጅ / የኃይል ምንጭ ጥቅም ላይ ያውሉ ነበር? |  | 1. መብራት 2. ኬሮሲን 3. ከሰል / እንጨት / ሣር 4. የእንስሳት ኩበት 5. ሌሎች |  |
| 608 | የመኖሪያ ቤቱን የውጭ ግድግዳ ለመሥራት ያገለግሉ ዋና ዋና ቁሳቁሶች ምንድ ናቸው? |  | 1. ድንጋይ በሲሚንቶ(ብሎኬት) 2. እንጨት በጭቃ 3. ድንጋይ በጭቃ 4. ሌሎች |  |
| 609 | የመኖሪያ ቤቱን ወለል ለመስራት ያገለገሉ ዋና ዋና ቁሳቁሶች ምንድን ናቸው? |  | 1. አፈር 2. ሊሾ 3. ሴራሚክ የተነጠፈ |  |
| 610 | የመኖሪያ ቤቱን ጣሪያ ለመሥራት ያገለግሉ ዋና ዋና ቁሳቁሶች ምንድ ናቸው? |  | 1. የቆርቆሮ ጣሪያ 2. የእንጨት ጣሪያ (የሳር ክዳን) 3. ሌሎች |  |
| 611 | የራስዎ የእርሻ መሬት ነበረዎት |  | 1. አዎ 2. የለም |  |
| 612 | መልስዎ አዎ ከሆነ ለ611 ስንት ሄክታር እርሻ መሬት ነበራችሁ? |  | 1. --------------ሄከ (የአከባቢ መለኪያ ይጠቀሙ) |  |
| 613 | ከሚከተሉት የእህል አይነቶች በዓመት ምንያህል ኩንታል ያገኙ ነበር |  | 1. ጤፍ-------------- 2. በቆሎ------------- 3. ስንዴ/ገብስ--------- 4. አተር/ባቄላ---------- |  |
| 614 | ቤተሰብዎ ከሚከተሉት ሀብቶች ውስጥ አንዱ ነበረዎት | 1. ሬዲዮ | 1. አዎ 2. የለም |  |
|  |  | 1. ሞባይል | 1. አዎ 2. የለም |  |
|  |  | 1. የዉሃ ፓምፕ | 1. አዎ 2. የለም |  |
|  |  | 1. አልጋ ከነፍራሹ(ስፖንጅ) | 1. አዎ 2. የለም |  |
|  |  | 1. ሶላር | 1. አዎ 2. የለም |  |
|  |  | 1. የፈረስ ጋሬ | 1. አዎ 2. የለም |  |
|  |  | 1. የልብስ ስፌት መኪን | 1. አዎ 2. የለም |  |
|  |  | 1. ሳይክል | 1. አዎ 2. የለም |  |
|  |  | 1. መብራት | 1. አዎ 2. የለም |  |
| 615 | ከዚህ ቤተሰብ አባላት ዉስጥ ከሚከተሉት የከብት እንስሳት አንዱ ነበራችሁ?( መልሱ አዎ ከሆነ ብዛቱን ያስቀምጡ) | 1. ላም | 1. አዎ---------------- 2. የለም |  |
|  |  | 1. ጥንድ በሬዎች | 1. አዎ -------------- 2. የለም |  |
|  |  | 1. ፈረስ | 1. አዎ----------------- 2. የለም |  |
|  |  | 1. በቅሎ | 1. አዎ----------------- 2. የለም |  |
|  |  | 1. አህያ | 1. አዎ------------------ 2. የለም |  |
|  |  | 1. ፍየል | 1. አዎ------------------ 2. የለም |  |
|  |  | 1. በግ | 1. አዎ------------------ 2. የለም |  |
|  |  | 1. የንፎ ቀፎ | 1. አዎ--------------- 2. የለም |  |
|  |  | 1. ዶሮ | 1. አዎ----------------- 2. የለም |  |
| 616 | ለመኝታ ምን ያህል ክፍሎች ያገለግላሉ ነበር? |  | ---------------------------- |  |
| 617 | ይህ ቤተሰብ የቤት አገልጋይ(ሎሌ/አበልተኛ) ነበረዉ? |  | 1. አዎ 2. የለም |  |
| 618 | የቤትዎ ገቢ በወር ስንት ነበር |  | --------------ብር |  |
| 619 | ባለፉት ሁለት አመታት በቤትዎ ዉስጥ የላም ወተት ኑሮዎት ያዉቃል |  | 1. አዎ 2. የለም |  |

**ስላዳመጡን እናመሰግናለን**
