# Supplementary material for: Time to breastfeeding cessation and its predictors among mothers who have children aged two to three years in Gozamin district, Northwest Ethiopia: A retrospective follow-up study
Source: PLoS One. 2022 Jan 21;17(1):e0262583. doi: 10.1371/journal.pone.0262583 (PMC8782324; doi:10.1371/journal.pone.0262583)
Supplement: Attachment — Submitted filename: BU Review BF Cessation for PLOS ONE 30 Sep 2021.docx [file pone.0262583.s003.docx]

**Title:**

Time to early breastfeeding cessation and its predictors among mothers who have children aged two to three years in Gozamin district, Northwest Ethiopia: a retrospective follow-up study

**Reviewer:**

Budi Utomo, Faculty of Public Health Universitas Indonesia, UI New Campus, Depok, West Java, Indonesia, Post code: 16424

Date: 30 September 2021

**Review results:**

This draft manuscript is about a community-based study of women who have children aged 2 to 3 years to assess incidence and predictors of breastfeeding cessation before 24 months in Gozamin District, Northwest Ethiopia.

The authors cited that the early breastfeeding cessation is a critical issue in Ethiopia it violates the WHO recommendation to breastfeeding the child up to 24 months and it caused 14000 preventable childhood deaths annually. However, the draft manuscript does not provide clear convincing evidence on the extent of breastfeeding cessation before 24 months on childhood death implications.

To convince, the breastfeeding duration important in preventing childhood illnesses, the authors cited the literature that children who ceased breastfeeding before 6 months (not before 24 months), as compared to the children who continued breastfeeding up to 24 months, have higher risk of morbidity and mortality. Hence, the cut-off of breastfeeding duration is at least 6 months, not 24 months used in this study/draft manuscript.

At the same time, however, the authors present statistics showing the breastfeeding cessation issue in Ethiopia is not more critical than the other countries, globally. The authors cited the percentage of children ceased breastfeeding before 24 months is 33% worldwide, 29% in Southern and Eastern African countries, while lower percentage in Ethiopia, 24%. Thus, the issue is less in Ethiopia than in other countries. Moreover, the overall mean of breastfeeding duration in Ethiopia is still quite long, 23 months, which is not far from the recommended breastfeeding duration of 24 months.

On the analysis, the study suffers from a lack of theory or conceptual framework on factors and their mechanisms to predicting the breastfeeding cessation. The study has not yet clearly conceptualized on what factors to be empirically tested in the analysis.

The study idea and objective should be revised and might focus on more critical breastfeeding issue, such as exclusive breastfeeding the first 6 months or the practice of too early feeding supplementation, appropriated to the Ethiopia context. The study should aalso develop a theory of factors predicting the breastfeeding behaviours. o0o
