# Supplementary material for: Time to breastfeeding cessation and its predictors among mothers who have children aged two to three years in Gozamin district, Northwest Ethiopia: A retrospective follow-up study
Source: PLoS One. 2022 Jan 21;17(1):e0262583. doi: 10.1371/journal.pone.0262583 (PMC8782324; doi:10.1371/journal.pone.0262583)
Supplement: Attachment — Submitted filename: Response to Reviewers.docx [file pone.0262583.s004.docx]

**Response to Reviewers**

**Author Feedback:** Dear editors and reviewers of this manuscript, we have no sufficient words to give our gratitude for your devotion of time and energy to improve the manuscript sufficient enough to be ready for production and then publication. As per your comments, we have tried to improve the manuscript and gave responses to those comments one by one. Dear editors and reviewers, even further we are ready to hear your comments and improve accordingly. Thanks a lot.

[Note: HTML markup is below. Please do not edit.]

Reviewers' comments:

Reviewer's Responses to Questions

**Comments to the Author**

1. Is the manuscript technically sound, and do the data support the conclusions?
The manuscript must describe a technically sound piece of scientific research with data that supports the conclusions. Experiments must have been conducted rigorously, with appropriate controls, replication, and sample sizes. The conclusions must be drawn appropriately based on the data presented.

Reviewer #1: Partly

Reviewer #2: Partly

**Author Response:** yes, it is sounded technically as all research procedures were implemented accordingly. To assure this the data set which is in Stata format (.DAT) was attached as a supportive file.

2. Has the statistical analysis been performed appropriately and rigorously?

Reviewer #1: I Don't Know

Reviewer #2: I Don't Know

**Author response:** yes, the appropriate and robust way to analyze time to event type of data was Cox regression, which is a popular and semiparametric type of modeling that nearly gives results obtained by parametric type of modeling in survival analysis at which data distribution was not well known. Hence, “time to breastfeeding cessation” is an outcome variable having a time-to-event nature that is handled by cox regression modeling with the assumptions of modeling were fulfilled. So the statistical analysis was performed using this modeling technique, it is appropriate.

3. Have the authors made all data underlying the findings in their manuscript fully available?

The [PLOS Data policy](http://www.plosone.org/static/policies.action#sharing) requires authors to make all data underlying the findings described in their manuscript fully available without restriction, with rare exceptions (please refer to the Data Availability Statement in the manuscript PDF file). The data should be provided as part of the manuscript or its supporting information, or deposited to a public repository. For example, in addition to summary statistics, the data points behind means, medians, and variance measures should be available. If there are restrictions on publicly sharing data—e.g. participant privacy or use of data from a third party—those must be specified.

Reviewer #1: Yes

Reviewer #2: Yes

**Author response:** Yes, all data used for this manuscript were obtained without any restriction and provided as a “supportive file” in the web application.

4. Is the manuscript presented in an intelligible fashion and written in standard English?

PLOS ONE does not copyedit accepted manuscripts, so the language in submitted articles must be clear, correct, and unambiguous. Any typographical or grammatical errors should be corrected at revision, so please note any specific errors here.

Reviewer #1: Yes

Reviewer #2: No

**Author response:** Thank you, we accept your comments. Typographical and grammatical errors in the revised manuscript were corrected and made to be clear.

5. Review Comments to the Author

Please use the space provided to explain your answers to the questions above. You may also include additional comments for the author, including concerns about dual publication, research ethics, or publication ethics. (Please upload your review as an attachment if it exceeds 20,000 characters)

**Reviewer #1: Major comments**

**Title**

You are using two terms (time and early) with similar meanings. I would suggest removing either of them. The title is not clearly stated. I suggest: Early breastfeeding cessation and its predictors among mothers with two to three years old children in Gozamin....

**Author response:** Accepted and modified as the time to breastfeeding cessation and its predictors among mothers who have children aged two to three years in Gozamin district, Northwest Ethiopia. As we all know the title should be precise, predictive, and well explanatory, which means that a title should have a minimum word that at least tell the nature of the outcome variable, the type of statistical model, and the design that will use. If we made the title “early breastfeeding cessation and its predictors……”, it tells us logistic regression and cross-sectional study design were used. Hence it was modified as “time to breastfeeding cessation and its predictors……”. In this case, it indicates that the outcome variable is “time to event” and hence the statistical modeling used was “survival analysis”.

Corresponding author: No need to mention these details here, put the adress next to your address above. To show the corresponding author, put * next to the name then mention what the * means. * Corresponding author

**Author response:** Accepted and corrected accordingly in the revised manuscript.

Abstract: What is the importance of mentioning the last sentence in the introduction? Delete it and add the aim/objective of your study

**Author response:** last two sentences in the introduction part of the abstract conclude that both statement of the problem and the objective of the study in a precise way. Of course, we modified it by adding the objective of the study in the revised manuscript to be more clear and attractive according to the journal requirement.

Methods (page 5)

I do not think that the you have used proper study design

**Author response:** The study aimed to detect the incidence of early breastfeeding cessation before the WHO recommendation and time to this cessation. For this purpose, we followed birth cohorts retrospectively they were born for the last two years with certain exposure statuses mentioned as variables. We identified mothers who have children aged two to three years of age and we took their exposure status and then we followed them until the outcome variable happened (time to breastfeeding cessation or the WHO recommended time=time to event or time to censoring as operationalized in the operational definition) by recalling of the mother and the records found at the Health post. Hence, a retrospective follow-up study was used in this way.

You have explained too much about study area which is less important, try to summarize in few sentences.

**Author response:** Thank you, accepted; and summarized accordingly in the revised manuscript.

What is your justification to take this timeframe October 2017 to September 20202?
**Author response:** This study was started at the end of September 2020. To follow mothers for two years (Follow up period), a one-year birth cohort was recruited (recruitment period). So the recruitment period started from October 1/2017 up to September 30/ 2018, and then we followed them from October 1/2018 up to September 30/2020, which was the follow-up period. That is why this time frame was taken.

The inclusion criteria is similar with the study population, try to summarize the subheadings and avoid repetition.

**Author response:** in the revised manuscript repetition was corrected and summarized within the single subheading as we accept your recommendation.

Exclusion criteria (last two lines), Not clear what you want to say? revise it

**Author response:** “Mothers who have not initially breastfed at least once for the child age two to three years now, and who gave care and breastfed other than their child” are the last two sentences in the exclusion criteria. Mothers who didn’t start breastfeeding for the child were excluded because they have no survival time (no breastfeeding duration). In the rural part of the Ethiopian context, some mothers care and breastfeed other than their own child, for example those children whose mothers were died may breastfeed the neighboring mothers breast milk and care by them. Such mothers were excluded from this study because we may not get the full survival time or censoring time during the follow-up time.

Sampling procedure: What is the total number of Kebele, from how much did you select ten?

**Author response:** There are 30 kebeles in the study area and 30% of the kebeles were taken randomly. Statistically, if 20% to 30% of the population were taken using the probability sampling technique, representativeness was maintained.

Why do you use the word "infant chaild pairs" Have you collect any data from the child? I would suggest to use mothers....

**Author response:** For this study, participants were Mother-child pairs because in our context some mothers become pregnant and give birth even within one year of the child. In this case, the mother should be paired with the older child whose age was two to three years, otherwise, they give a response to the latest child. To avoid confusion for the data collectors and participants, mother-child pairs were used.

data collection: I do not know why you have taken date of birth as starting point? it is obvious. Your dependent variable is early BF cessation before 24 months and the participants are mothers with children 2-3 years. So why are you using retrospective follow up design? which is used to assess the outcome of certain exposure. I think the design you have used is not correct. I am ready to hear your explanation about this. The last three lines of page 8: To answer this, why do you use retrospective follow up? because it is obvious that they have started after birth then stopped in one specific time.

**Author response**: The dependent variable is “time to breastfeeding cessation” not only early breastfeeding cessation. The study aims to detect the survival time of breastfeeding up to WHO recommendation. So, to get the survival time the starting point of follow-up was birth (breastfeeding starts immediately after birth, nearly 30 minutes) and followed mother-child pairs until event or censoring. The exposure variables were identified during birth and then follow mother-child pairs until event or censoring. That is why a retrospective follow-up study design was used.

Data collection tool: This should come before data collection. I would suggest to merge with the above sub headings. What was the findings of your pretest, summarize in one or two sentences? was there any problem with the tool? what do you find from your pretest?

**Author response:** As per your recommendation, subheadings (Data collection procedures and data collection tools) were merged as Data collection tools and procedures in the revised manuscript. Findings of pretest were summarized in the revised manuscript as “not easily understandable questionnaires’ were modified and some variables missed during pretest were incorporated in the final questionnaires”.

Results

First sub title: Why are you referring as baseline? it is confusing. do you have any data taken during follow up? I think such category is used for multiphase study but yours is single phase study. Therefore, better to sat Sociodemographic characteristics
Fourth line of first sub heading: Did you had any follow up time in your study? I this the main problem is related to the study design. What is the importance of mentioning this as incidence?
**Author response:**  In this study, initially we took baseline maternal characteristics such as educational status, marital status, age, etc. of the mother at childbirth, and even if it is not a fully multi-phasing study, we took follow up data from the mother and other records such as immunization certificate, starting time of complementary feeding, presence of cow milk at home at least for the last two seasons. We didn’t take further sociodemographic characteristics after birth. Due to this “baseline sociodemographic characteristics” were used as a subheading. Another point you raised was “follow-up time”. It is a follow-up study from birth up to date of breastfeeding cessation or censoring time to get the survival time of breastfeeding duration. By this follow-up time, the new occurrence of breastfeeding cessation was counted and calculated the incidence rate of breastfeeding cessation before the WHO recommendation time. That is why “incidence” was written in the manuscript.

Minor comments

Title page: Be consistent, you have added affiliation to 2 and 3 but 1 and 4 did not have affiliation. Put each email next to their address

**Author response:** as per comments the title page consistency was revised (see the revised manuscript with track change).

Abstract: add comma to 14000, in the conclusion you have used different line spacing
data collection procedure there is a word "For 4 data collectors.... What? not clear
There are grammatical errors, the language need revision

**Author response:** all minor comments in the initial manuscript were modified in the revised manuscript including grammatical and language errors. For “4 data collectors” means that for interviewing mother-child pairs 2 B.Sc. nurses and 2 Health officers were involved, a total of four data collectors for this purpose. It is revised.

Reviewer #2: The draft manuscript should convince clearly with evidence the breastfeeding cessation is a critical issue in Ethiopia. Otherwise, the analyses objective should be revised to be appropriated with the country evidence-based breastfeeding issue. There is a need for the study to develop a theory of what factors and their mechanisms to predicting the breastfeeding behaviour. The arguments provided in the background is often inconsistent (See my attached written review).

**Author response:** Dear reviewer #2, thank you for your valuable comments and your devotion of time to this manuscript to be a full scientific paper. All scientific papers distributed to the scientific community should convince readers and be part of the solution for the problem identified in the research. This study identified that breastfeeding cessation before the WHO recommendation time was a problem that leads to 14000 childhood deaths and 5 million diarrheal and pneumonia cases annually in Ethiopia. As you explained, it is not only due to exclusive breastfeeding cessation but includes breastfeeding cessation later to 6 months that caused this problem. The proportion of Breastfeeding cessation before WHO recommended duration was less than in Ethiopia as compared to the overall sub-Saharan African countries, but the duration of breastfeeding in Ethiopia was in a decreasing trend from 2005 EDHS to 2016 EDHS (92%to 85% to 76%) with regional variation (EDHS 2016). This increased the health care costs of Ethiopia up to 0.003% GNI of the country. So that identifying whether breastfeeding cessation timing was a problem or not specifically in the rural part of Northwest Ethiopia has paramount importance. Another issue you raised is that “there is no conceptual framework of factors how that cause timing variation in breastfeeding cessation”. The original draft of this study has a conceptual framework that shows the relation between independent variables with the dependent variable, but it is not attached in the manuscript due to the journal requirement. (For further, you can see below)

Conceptual framework

Time to breastfeeding cessation

Sociodemographic factors

- Age of the mother
- Marital status of the mother
- Educational status
- Occupation
- Wealth Index
- Number of children

Nutritional related factors

- Starting time of complementary feeding
- Use of formula milk
- Presence of cow milk at household

Obstetric and HSU Related factors

- Antenatal care follow up
- Mode of delivery
- Place of delivery
- Immunization follow up
- Breastfeeding experience

6. PLOS authors have the option to publish the peer review history of their article ([what does this mean?](https://journals.plos.org/plosone/s/editorial-and-peer-review-process#loc-peer-review-history)). If published, this will include your full peer review and any attached files.


If you choose “no”, your identity will remain anonymous but your review may still be made public.


**Do you want your identity to be public for this peer review?** For information about this choice, including consent withdrawal, please see our [Privacy Policy](https://www.plos.org/privacy-policy).

Reviewer #1: No

Reviewer #2: No

**Author response:** No

[NOTE: If reviewer comments were submitted as an attachment file, they will be attached to this email and accessible via the submission site. Please log into your account, locate the manuscript record, and check for the action link "View Attachments". If this link does not appear, there are no attachment files.]

While revising your submission, please upload your figure files to the Preflight Analysis and Conversion Engine (PACE) digital diagnostic tool, <https://pacev2.apexcovantage.com/>. PACE helps ensure that figures meet PLOS requirements. To use PACE, you must first register as a user. Registration is free. Then, login and navigate to the UPLOAD tab, where you will find detailed instructions on how to use the tool. If you encounter any issues or have any questions when using PACE, please email PLOS at [figures@plos.org](mailto:figures@plos.org). Please note that Supporting Information files do not need this step.
