# Supplementary material for: Time to breastfeeding cessation and its predictors among mothers who have children aged two to three years in Gozamin district, Northwest Ethiopia: A retrospective follow-up study
Source: PLoS One. 2022 Jan 21;17(1):e0262583. doi: 10.1371/journal.pone.0262583 (PMC8782324; doi:10.1371/journal.pone.0262583)
Supplement: Attachment — Submitted filename: Response to Reviewers.docx [file pone.0262583.s005.docx]

**Response to Reviewers**

**Author Feedback:** Dear editors and reviewers of this manuscript, we have no sufficient words to give our gratitude for your devotion of time and energy to improve the manuscript sufficient enough to be ready for production and then publication. As per your comments, we have tried to improve the manuscript and gave responses to those comments one by one. Dear editors and reviewers, even further we are ready to hear your comments and improve accordingly. Thanks a lot.

[Note: HTML markup is below. Please do not edit.]

**Editors’ comments:** Thank you for addressing the reviewers' comments and suggestions. However, I cannot recommend acceptance of the manuscript yet because your marked-up copy of the revised version does not have track changes on it, which makes it difficult to compare the changes you have made against the original draft. Therefore, please resubmit the revised version with track changes on. Further, please also insert the conceptual framework in the text so that I can have it reviewed.

**Authors' response:** Thank you for your valuable comments and suggestions. As per your comments, we have tried to add a copy of the marked up or tracked change manuscript. Besides, we have added a conceptual framework on the manuscript with a brief text form and the full form is attached as figure -1 (Further see page 4 paragraph 2 of the manuscript).

Reviewers' comments:

Reviewer #2: The draft manuscript should convince clearly with evidence the breastfeeding cessation is a critical issue in Ethiopia. Otherwise, the analyses objective should be revised to be appropriated with the country evidence-based breastfeeding issue. There is a need for the study to develop a theory of what factors and their mechanisms to predicting the breastfeeding behaviour. The arguments provided in the background is often inconsistent (See my attached written review).

**Author response:** Dear reviewer #2, thank you for your valuable comments and your devotion of time to this manuscript to be a full scientific paper. All scientific papers distributed to the scientific community should convince readers and be part of the solution for the problem identified in the research. This study identified that breastfeeding cessation before the WHO recommendation time was a problem that leads to 14000 childhood deaths and 5 million diarrheal and pneumonia cases annually in Ethiopia. As you explained, it is not only due to exclusive breastfeeding cessation but includes breastfeeding cessation later to 6 months that caused this problem. The proportion of Breastfeeding cessation before WHO recommended duration was less than in Ethiopia as compared to the overall sub-Saharan African countries, but the duration of breastfeeding in Ethiopia was in a decreasing trend from 2005 EDHS to 2016 EDHS (92%to 85% to 76%) with regional variation (EDHS 2016). This increased the health care costs of Ethiopia up to 0.003% GNI of the country. So that identifying whether breastfeeding cessation timing was a problem or not specifically in the rural part of Northwest Ethiopia has paramount importance. Another issue you raised is that “there is no conceptual framework of factors how that cause timing variation in breastfeeding cessation”. The original draft of this study has a conceptual framework that shows the relation between independent variables with the dependent variable. Now, it is attached in the manuscript as per your comments. (For further, you can see at the manuscript-page 4 paragraph 2 and figure 1).

[NOTE: If reviewer comments were submitted as an attachment file, they will be attached to this email and accessible via the submission site. Please log into your account, locate the manuscript record, and check for the action link "View Attachments". If this link does not appear, there are no attachment files.] While revising your submission, please upload your figure files to the Preflight Analysis and Conversion Engine (PACE) digital diagnostic tool, <https://pacev2.apexcovantage.com/>. PACE helps ensure that figures meet PLOS requirements. To use PACE, you must first register as a user. Registration is free. Then, login and navigate to the UPLOAD tab, where you will find detailed instructions on how to use the tool. If you encounter any issues or have any questions when using PACE, please email PLOS at [figures@plos.org](mailto:figures@plos.org). Please note that Supporting Information files do not need this step.
