# Supplementary material for: Time to breastfeeding cessation and its predictors among mothers who have children aged two to three years in Gozamin district, Northwest Ethiopia: A retrospective follow-up study
Source: PLoS One. 2022 Jan 21;17(1):e0262583. doi: 10.1371/journal.pone.0262583 (PMC8782324; doi:10.1371/journal.pone.0262583)
Supplement: Attachment — Submitted filename: Response to Reviewers.docx [file pone.0262583.s007.docx]

**Response to Reviewers**

**Author Feedback: Dear editors and reviewers of this manuscript, we have no sufficient words to give our gratitude for your devotion of time and energy to improve the manuscript sufficient enough to be ready for production and then publication. As per your comments, we have tried to improve the manuscript and gave responses to those comments one by one. Dear editors and reviewers, even further we are ready to hear your comments and improve accordingly. Thanks a lot.**

[Note: HTML markup is below. Please do not edit.]

**Editors’ comments:** Thank you for uploading the revised manuscript with track changes on, which has made it possible for me to compare your responses with the reviewers' comments. However, minor editorial omissions continue to remain in Revision#3. For example, in the Introduction to the Abstract you have cited the number 14000 without a comma as a ,000 separator. Please write the number as 14,000. Similarly, in Methods in the Abstract you have started the sentence with a lower case "a" such as "a community-based retrospective follow-up study was used...." . This should be written as "A community-based retrospective follow-up study was used....". (i.e. beginning with an upper case "A"). PLOS One does not provide copy editing, therefore please make the corrections as indicated above, and have the entire revised manuscript thoroughly edited for English.

**Authors' response: Thank you for your valuable comments and suggestions. As per your comments, we have tried to improve typological and grammatical problems on the revised manuscript.**
